# A meta-analysis of CAG (cytarabine, aclarubicin, G-CSF) regimen for the treatment of 1029 patients with acute myeloid leukemia and myelodysplastic syndrome

**DOI:** 10.1186/1756-8722-4-46

**Published:** 2011-11-14

**Authors:** Guoqing Wei, Wanmao Ni, Jen-wei Chiao, Zhen Cai, He Huang, Delong Liu

**Affiliations:** 1 Bone Marrow Transplantation Center, the First Affiliated Hospital, Zhejiang University School of Medicine, Hangzhou, China; 2 Division of Hematology and Oncology, New York Medical College and Westchester Medical Center, Valhalla, New York, USA

**Keywords:** acute myeloid leukemia, myelodysplastic syndrome, CAG, aclarubicin, meta-analysis

## Abstract

The regimen of cytarabine, aclarubicin and G-CSF (CAG) has been widely used in China and Japan for treatment of acute myeloid leukemia (AML) and myelodysplastic syndrome (MDS). We searched literature on CAG between 1995 and 2010 and performed a meta-analysis to determine its overall efficacy using a random-effects or fixed-effects model. Thirty five trials with a total of 1029 AML (n = 814) and MDS (n = 215) patients were included for analysis. The CR rate of AML (57.9%) was significantly higher than that of MDS (45.7%) (p < 0.01). No difference in CR was noted between the new (56.7%) and relapsed/refractory AML (60.1%) (p > 0.05). The CR rate was also significantly higher in patients with favorable (64.5%) and intermediate (69.6%) karyotypes than those with unfavorable one (29.5%) (p < 0.05). Remarkably, the CR rate of CAG was significantly higher than those of non-CAG regimens (odds ratio 2.43). CAG regimen was well tolerated, with cardiotoxicity in 2.3% and early death in 5.2% of the cases. In conclusion, CAG regimen was an effective and safe regimen for the treatment of AML, and may be more effective than non-CAG regimens. Randomized controlled trials are strongly recommended to evaluate its efficacy and safety in comparison with the current standard treatment.

## Introduction

Intensive chemotherapy can achieve complete remission (CR) in 60% - 80% of patients with newly diagnosed *de novo *acute myeloid leukemia (AML) [1,2]. However, current therapy is still unsatisfactory in patients with high-risk AML including elderly, relapsed, refractory, and secondary AML. Intensive chemotherapy is generally unsatisfactory in these patients because of drug resistance, poor performance status (PS), dysfunction of multiple organs, and high treatment-related toxicities, leading to high early death (ED) rate [3-6]. Novel agents and regimens are being developed for this group of patients [2,7-10].

Aclarubicin is an oligosaccharide anthracycline, and an antineoplastic antibiotic. It can intercalate into DNA and interact with topoisomerase I and II, thereby inhibiting DNA replication and DNA repair [11]. This agent is less cardiotoxic than doxorubicin and daunorubicin [12,13]. In 1995, a Japanese group reported a new chemotherapy regimen for AML treatment, which integrated granulocyte colony-stimulating factor (G-CSF) priming into the combination of low-dose cytarabine (Ara-C) and aclarubicin (CAG regimen) [14]. The CAG regimen consists of low-dose Ara-C 10 mg/m^2^, SQ Q12 hr on days 1-14, aclarubicin 7 mg/m^2^, QD on days 1-8, or 14 mg/m^2^, IV QD on days 1-4, and G-CSF 200 μg/m^2^, SQ QD on days 1-14. The rationale for the regimen includes: (1) G-CSF priming has been found to preferentially potentiate Ara-C and anthracycline- mediated cytotoxicity on AML cells and AML progenitor cells (CFU-AML), presumably by enhancing G0 resting AML cells into the cell cycle [15]; (2) prolonged exposure to low-dose Ara-C and G-CSF can lead to preferential killing of CFU-AML [16]; (3) Aclarubicin is effective regardless of multi-drug resistance gene status [17]; (4) CAG combination may inhibit the self-renewal capacity of CFU-AML and leukemia stem cells [18]. Since then, CAG regimen has been used to treat AML and myelodysplastic syndrome (MDS) patients widely, particularly for high-risk and elderly patients, in China and Japan. However, the overall efficacy and safety of CAG regimen have not been adequately evaluated. All published studies on CAG were small phase II studies with significant variation in clinical outcomes. In this study, we performed a systematic review and meta-analysis to assess the overall treatment efficacy and the adverse events of the CAG regimen.

## Materials and methods

### Data source

The databases of PubMed, Wanfang Data, as well as American Society of Hematology (ASH) and American Society of Clinical Oncology (ASCO) annual meeting abstracts were searched for articles published in English, Chinese and Japanese languages between January 1995 and December 2010. Eligible studies were relevant clinical trials on AML and MDS patients treated with CAG regimen. Key words used were *CAG, chemotherapy, leukemia, and MDS*. An independent search using the citation database Wanfang Data (http://www.wanfangdata.com) was also performed to identify those publications in Chinese only.

### Study selection

The publications identified were carefully screened. Preclinical studies, case reports and reviews were excluded. Several reports had duplicate or overlapping information. Only the latest updated reports were included for meta-analysis. For two studies by Qian's group [19,20], it was impossible to decipher whether duplicate or overlapping information was used. These were treated as individual studies. Efforts also were made to contact the Chinese investigators to clarify study issues. We also contacted Japanese investigators to obtain original publications in Japanese with English abstracts.

### Clinical endpoints

We extracted details on study characteristics, patient characteristics, treatment information, results and follow-up from the selected trials. Two investigators reviewed the data independently (GW and DL). The primary end point of the meta-analysis was CR rate. AML CR was defined using the criteria developed by an International Working Group [21]. The criteria for refractory and relapsed AML were described previously [22]. The criteria for karyotype classification have evolved over the past decades [4,23,24]. In general, the 35 studies chosen for final analysis followed the standard definitions described above. CR rates and side effects were carefully reviewed and compiled. Two major side effects, cardiotoxicity and early death (ED), were chosen for further analysis since these two toxicities are generally more objective. In studies which did not clearly define the criteria of cardiotoxicity, the toxicity was counted as being present when a study reported mortality cases due to cardiac causes. There were different criteria for ED definition among the studies. All deaths of any cause within 8 weeks of induction therapy were counted as ED in this meta-analysis since most of the early death reports in this series were within 8 weeks of induction therapy. This would also reduce the chance of under-reporting the toxicity. Standard age definitions were used, *i.e*. young AML: age < = 60; Elderly AML: age >60.

### Statistical analysis

All statistical analyses were performed using version 2 of the Comprehensive MetaAnalysis program (Biostat, Englewood, NJ, USA). The CR rates of patients treated with CAG regimen were directly extracted from individual studies. For subgroup analysis of patients with newly diagnosed, refractory/relapsed AML, and MDS or AML transformed from MDS (MDS/t-AML) patients, numbers of patients in CR were extracted from individual studies and CR rates were recalculated from the derived data. For studies with a control group, the odds ratio (OR) of CR rates was also calculated. For the meta-analysis, both fixed-effects and random-effects models were considered. For each meta-analysis, the Cochrane's Q statistic was first calculated to assess the heterogeneity of the included studies. For p values less than 0.1, the assumption of homogeneity was deemed invalid, and the random-effects model was used only after substantial efforts were made to explore the possible reasons for the heterogeneity. Otherwise, data were assessed using both fixed-effects and random-effects models. We used the Begg and Egger tests to evaluate the presence of publication bias regarding the primary end point CR rates. A two-tailed p value of less than 0.05 was deemed statistically significant. All the statistical analyses were done by GW and DL.

## Results

### Selection of studies

**Figure 1:**
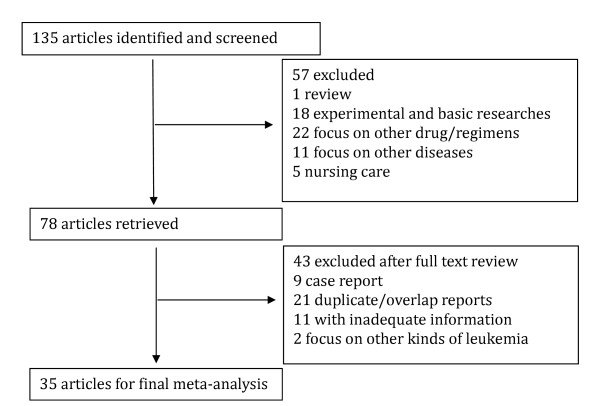
**Flow chart for study selection in the meta-analysis**.

Using the key words, our search yielded a total of 135 studies on CAG. Fifty seven studies were irrelevant to CAG regimen trials, and were excluded. Another 32 studies were eliminated due to inadequate information, duplicate and/or overlap reporting. Nine case reports and 2 studies focus on other kinds of leukemia were also excluded. A total of 35 clinical studies were eligible for inclusion and were used for final meta-analysis (Figure 1).

### Characteristics of studies included in the meta-analysis

Thirty five trials were included in the present analysis, with a total of 1029 patients accrued. Characteristics of the 35 trials are listed in Tables 1 and 2. Twenty three of the studies were on AML. Six of the studies focused on MDS. The rest 6 studies enrolled both AML and MDS patients. A total of 814 AML patients were accrued in 29 studies. Among the 814 AML patients, 327 patients had newly diagnosed AML, 370 patients were relapsed/refractory (R/R) AML. AML status of 117 patients was not specified in 7 studies [19,20,25-29]. The age varied widely, ranging from 15 to 88 years old. Two studies did not indicate the age range (Table 1). Unfortunately, median age was not specified in most studies. Among the 814 patients, 367 were elderly AML patients (45.1%). Cytogenetic characteristics from 203 AML patients were reported in 6 studies. These patients were grouped into 3 categories according to the karyotypes: favorable, intermediate and unfavorable. CAG regimen was compared to historical controls using non-CAG induction regimens in 7 studies with 327 patients. The number of CAG cycles was clearly reported in 17 studies involving 397 patients, of whom 144 (36.2%) were induced twice with CAG.

**Table 1:** Efficacy, cardiotoxicity and early death rate of CAG in AML


**Study (ref)**	**Year**	**No**. Patients *	**Median age (range)**	**Aclarubicin dosage**	**AML No**.	**AML CR No**.	**AML CR %**	**OS (m) (range)**	**CT No**.	**ED No**.

Yamada [14]	1995	18	44 (18-74)	10-14 mg/m^2 ^× 4d	18	15	83	17 (2-28)	0	0

Saito [35]	1995	18	NR(18-74)	10 or 14 mg/m^2 ^× 4d	18	15	83	15 (1-35)	1	0

Saito [36]	1996	28	NR(18-74)	10 or 14 mg/m^2 ^× 4d	28	24	86	17 (1-47)	0	0

Tabata [37]	1998	76	NR(60-83)	14 mg/m^2 ^× 4d	8	5	63	NA	NA	NA

Saito [38]	2000	69	51 (15-82)	10 or 14 mg/m^2 ^× 4d	51	31	62	NA	0	0

Hirayama [39]	2003	18	NR(65-80)	14 mg/m^2 ^× 4d	9	6	67	9 (1-43)	0	1

Li [26]	2005	112	47 (15-81)	14 mg/m^2 ^× 4d **o**r 8 mg/m^2 ^× 8d	99	44	44	NA	0	1

Kong [40]	2005	20	NR(18-80)	10-14 mg/m^2 ^× 4d	20	11	55	NA	0	0

Yang [41]	2005	16	NR(60-82)	6 mg/m^2 ^× 8d	16	9	56	NA	0	1

Huang [25]	2005	30	NR(15-67)	5-7 mg/m^2 ^× 8d	30	22	73	NA	0	1

Qian [19]	2005	21	NR(15-81)	10 mg/d × 8d	21	14	67	NA	0	2

Xie [42]	2006	25	NR(55-78)	10 mg/m^2 ^× 4d	25	12	48	NA	0	3

Liu [27]	2006	13	NR(18-77)	10-14 mg/m^2 ^× 4d	13	5	39	NA	0	0

Wang [43]	2006	11	NR(25-72)	10 mg/m^2 ^× 8d	11	4	36	NA	0	0

Wu [44]	2007	15	NR(60-84)	7 mg/m^2 ^× 8d	15	8	53	NA	1	1

Qian [20]	2007	50	65 (60-81)	10 mg/d × 8d	50	29	58	14 (1-60)	0	4

Su [29]	2007	16	NR	20 mg/d × 4d	16	9	56	NA	0	0

Guo [45]	2007	8	NR(18-56)	5-7 mg/m^2^×5d	8	4	50	NA	0	0

Chen [46]	2008	75	NR(60-85)	10 mg/d × 8d	34	23	68	NA	0	0

Sang [47]	2008	45	NR(60-73)	7 mg/m^2 ^× 8d	23	9	39	NA	0	1

Bian [48]	2008	46	NR(18-72)	10-14 mg/m^2 ^× 4d	26	20	77	NA	0	0

Chai [49]	2009	17	NR	15 mg/m^2^×7d	17	8	47	NA	0	0

Zhu [50]	2009	50	NR(15-69)	10 mg/d × 8d	30	14	47	NA	0	1

Ni [51]	2009	70	NR(22-85)	14 mg/m^2 ^× 4d	61	34	56	28 (1-89)	0	3

Feng [52]	2010	32	NR(60-74)	5-7 mg/m^2 ^× 8d	16	9	56	NA	0	0

Ma [28]	2010	31	NR(19-71)	14 mg/m^2 ^× 4d	26	14	54	NA	0	0

Li [53]	2010	38	NR(19-61)	5-7 mg/m^2 ^× 8d	18	14	78	NA	0	0

Liang [54]	2010	54	NR(17-82)	5-7 mg/m^2 ^× 8d	39	21	54	NA	1	7

Suzushima [55]	2010	68	76 (60-88)	14 mg/m^2 ^× 4d	68	33	49	9 (0-56)	NA	15

Abbreviations: AML: acute myeloid leukemia; Ref: references; NA: not available; NR: not reported; *: total number of AML and MDS patients; m: month; CR: complete remission; OS: overall survival; CT: cardiotoxicity; ED: early death; CAG: cytarabine, aclarubicin and G-CSF.

**Table 2:** Efficacy, cardiotoxicity and early death rate of CAG in MDS/t-AML


**Study (ref)**	**Year**	**No**. Patients *	**Median age (range)**	**Aclarubicin dosage**	**MDS/t-AML No**.	**CR No**.	**CR %**	**CT No**.	**ED No**.

Saito [38]	2000	69	51 (15-82)	10 or 14 mg/m^2 ^× 4d	18	8	44	0	0

Li [26]	2005	112	47 (15-81)	14 mg/m^2 ^× 4d or 8 mg/m^2 ^× 8d	13	5	39	0	0

Sui [56]	2008	17	NR(26-73)	10-14 mg/m^2 ^× 4d	17	6	35	0	0

Jin [57]	2008	14	NR(38-78)	10-14 mg/m^2 ^× 4d	14	6	43	0	0

Deng [58]	2008	39	NR(52-78)	5-6 mg/m^2^×7d	16	9	55	0	0

Su [59]	2009	33	60 (28-77)	10 mg/d × 8d	33	14	42	1	0

Ni [51]	2009	70	NR(22-85)	14 mg/m^2 ^× 4d	9	5	56	0	1

Ma [28]	2010	31	NR(19-71)	14 mg/m^2 ^× 4d	5	3	60	0	0

Li [60]	2010	20	NR(36-74)	5-7 mg/m^2 ^× 8d	20	9	45	0	0

Zhu [61]	2010	46	54 (31-72)	10 mg/m^2 ^× 8d	28	13	46	0	0

Chen [62]	2010	27	NR(21-72)	10 mg/d × 8d	27	15	56	0	0

Liang [54]	2010	54	NR(17-82)	5-7 mg/m^2 ^× 8d	15	5	33	0	2

Abbreviations: AML: acute myeloid leukemia; MDS: myelodysplastic syndrome; MDS/tAML: MDS or MDS transformed AML; Ref: references; NR: not reported; *: total number of AML and MDS patients; CR: complete remission; OS: overall survival; CT: cardiotoxicity; ED: early death; CAG: cytarabine, aclarubicin and G-CSF.

### Publication bias

No evidence of publication bias was detected for the primary end point, CR, of this study by either the Begg or Egger test (Begg test, p = 0.35; Egger test, p = 0.15).

### Efficacy of CAG regimen for all AML and high-risk MDS/transformed AML

**Figure 2:**
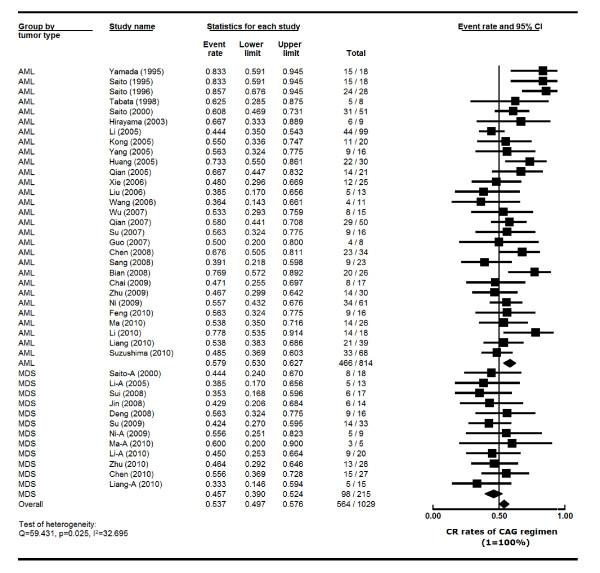
**Comparison of CR rates of CAG regimen in AML and MDS patients--Forest plot of CR event rates**. Summary CR rates of CAG regimen were calculated using the random-effects model. Horizontal lines through the squares represent 95% CIs. The diamonds represent the overall CR event rate from the meta-analyses and the corresponding 95% CIs. The studies that enrolled both AML and MDS were separated into two groups for this analysis, indicated by "-A". **Abbreviations**: AML: acute myeloid leukemia; MDS: myelodysplastic syndrome; CI: confidence interval; CR: complete remission; CAG: cytarabine, aclarubicin and G-CSF.

The heterogeneity test of CR event rates from the 35 studies revealed Q 59.431, p 0.025, I^2 ^32.695, indicating the CR event rates were highly variable. Therefore, the CR event rates were calculated using the random-effects model (Figure 2). The overall CR rate for the 1029 patients was 53.7% (95% CI, 49.7%-57.6%). Data available from 29 trials with 814 AML patients showed that the CR rate was 57.9% (95% CI, 53.0%-62.7%). As for the 215 patients with MDS and transformed AML (MDS/t-AML) from 12 trials, the CR rate was 45.7% (95% CI, 39.0%-52.4%) (Figure 2).

We also compared CAG in AML versus MDS. The higher CR rate in AML (57.9%) than in MDS (45.7%) was statistically significant (p = 0.004) (Figure 2). This is in agreement with the past observations that AML responds better to chemotherapy in general than MDS.

Among AML patients, the median OS was 15 months (range 9-28 months). The number of CAG cycles was specified in 17 studies involving 397 patients, of whom 144 (36.2%) were induced twice with CAG. Of the patients who received one cycle, 46.60% (185/397) went into CR, another 8.31% (33/397) of the patients achieved CR after re-induction with CAG.

### Efficacy of CAG regimen for newly diagnosed vs relapsed/refractory AML

**Figure 3:**
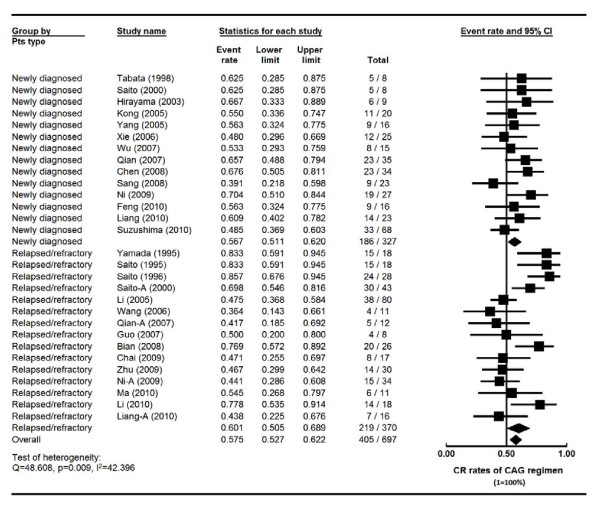
**Comparison of complete remission (CR) rates of CAG regimen in new, refractory and relapsed AML patients--Forest plot of CR event rates**. Summary CR rates of CAG regimen were calculated using the random-effects model. Horizontal lines through the squares represent 95% confidence interval (CI). The diamonds represent the overall CR event rate from the meta-analyses and the corresponding 95% CIs. The studies that enrolled both new and relapsed/refractory AML were separated into two groups for this analysis, indicated by "-A". **CAG**: cytarabine, aclarubicin and G-CSF.

There were a total of 327 new AML patients from 14 studies, and 370 patients with relapsed/refractory (R/R) AML from 15 studies. Four of these studies enrolled both new and R/R AML (Table 3 and Figure 3). These four studies were separated into new and R/R AML groups for meta-analysis. Another seven studies did not specify AML status of the rest 117 patients. These 117 patients were therefore excluded for this comparison (Table 3). The heterogeneity test of CR event rates of these studies revealed Q 48.608, p 0.009, I^2 ^42.396, indicating the CR event rates were highly variable. Therefore, the CR event rates were calculated using the random-effects model (Figure 3). The CR rate for the newly diagnosed AML patients was 56.7% (95% CI, 51.1%-62.0%). The CR rate of the 370 R/R AML patients was 60.1% (95% CI, 50.5%-68.9%) (Figure 3). Interestingly, no significant difference in CR rate was noted between the newly diagnosed and R/R AML patients (p = 0.539) (Table 3 and Figure 3), suggesting that this novel regimen may overcome AML resistance.

**Table 3:** Efficacy of CAG in newly diagnosed, relapsed and refractory AML


**Study (ref)**	**Year**	**Newly diagnosed AML**			**R/R AML**		
	
		**No**. **Patients**	**CR No**.	**CR %**	**No**. **Patients**	**CR No**.	**CR %**

Yamada [14]	1995	NA	NA	NA	18	15	83

Saito [35]	1995	NA	NA	NA	18	15	83

Saito [36]	1996	NA	NA	NA	28	24	86

Tabata [37]	1998	8	5	63	NA	NA	NA

Saito [38]	2000	8	5	63	43	30	70

Hirayama [39]	2003	9	6	67	NA	NA	NA

Li [26]	2005	NA	NA	NA	80	38	47

Kong [40]	2005	20	11	55	NA	NA	NA

Yang [41]	2005	16	9	56	NA	NA	NA

Xie [42]	2006	25	12	48	NA	NA	NA

Wang [43]	2006	NA	NA	NA	11	4	36

Wu [44]	2007	15	8	53	NA	NA	NA

Qian [20]	2007	35	23	66	12	5	40

Guo [45]	2007	NA	NA	NA	8	4	50

Chen [46]	2008	34	23	68	NA	NA	NA

Sang [47]	2008	23	9	39	NA	NA	NA

Bian [48]	2008	NA	NA	NA	26	20	77

Chai [49]	2009	NA	NA	NA	17	8	47

Zhu [50]	2009	NA	NA	NA	30	14	46

Ni [51]	2009	27	19	70	34	15	44

Feng [52]	2010	16	9	56	NA	NA	NA

Ma [28]	2010	NA	NA	NA	11	6	55

Li [53]	2010	NA	NA	NA	18	14	78

Liang [54]	2010	23	14	61	16	7	44

Suzushima [55]	2010	68	33	49	NA	NA	NA

Abbreviations: AML: acute myeloid leukemia; MDS: myelodysplastic syndrome; Ref: references; NA: not available; m: month; CR: complete remission; OS: overall survival; CAG: cytarabine, aclarubicin and G-CSF.

### CR rates in AML patients according to karyotypes

**Figure 4:**
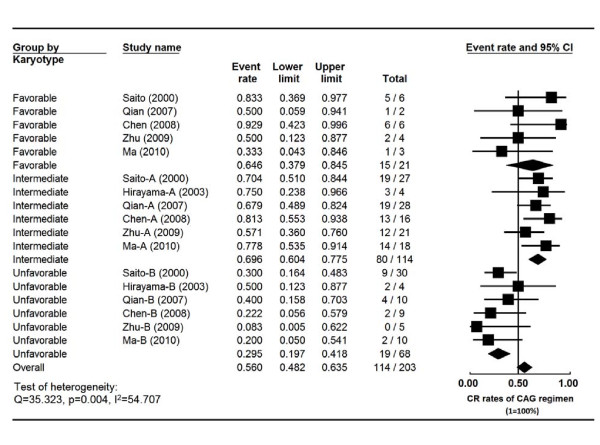
**Comparison of the CR rates of CAG regimen in AML patients according to karyotypes**. Summary CR rates of CAG regimen were calculated using the random-effects model. Horizontal lines through the squares represent 95% CIs. The diamonds represent the overall CR event rate from the meta-analyses and the corresponding 95% CIs.

Cytogenetic characteristics from 203 AML patients were reported in 6 studies (Table 4). The heterogeneity test of CR event rates revealed Q 35.323, p 0.004, I^2 ^54.707, indicating the CR event rates were highly variable. Therefore, the CR event rates were calculated using the random-effects model (Figure 4). The CR rates were 64.5% (95% CI, 38.8%-83.9%), 69.6% (95% CI, 60.4%-77.5%) and 29.5% (95% CI, 19.7%-41.8%) in favorable, intermediate and unfavorable groups, respectively. Statistical difference of the CR rates was noted between the favorable and unfavorable groups (p = 0.018) as well as between the intermediate and unfavorable groups (p < 0.001). No significant difference was present between the favorable and intermediate groups (p = 0.705) (Figure 4).

**Table 4:** Efficacy of CAG regimen in AML patients according to karyotypes


**Study**	**Year**	**Total No**. **patients**	**No. Patients**			**No. CR**			**CR %**		
			
			**Favorable**	**Intermediate**	**Unfavorable**	**Favorable**	**Intermediate**	**Unfavorable**	**Favorable**	**Intermediate**	**Unfavorable**

Saito [38]	2000	63	6	27	30	5	19	9	83	70	29

Hirayama [39]	2003	9	1	4	4	1	3	2	100	75	50

Qian [20]	2007	40	2	28	10	1	19	4	50	68	40

Chen [46]	2008	34*	6	16	9	6	13	2	100	81	22

Zhu [50]	2009	30	4	21	5	2	12	0	50	57	0

Ma [28]	2010	31	3	18	10	1	14	2	33	78	20

Abbreviation: AML: acute myeloid leukemia; CR: complete remission; CAG: cytarabine, aclarubicin and G-CSF; *: karyotyping from 3 patients was indeterminate.

### CAG vs non-CAG regimens for AML induction

**Figure 5:**
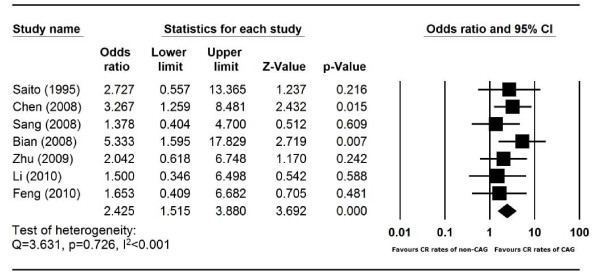
**Comparison of the CR rates of CAG regimen and non-CAG regimens in AML patients**. Summary Odds ratio (OR) of CR rates of CAG regimen versus non-CAG regimen was calculated using the fixed-effects model. Horizontal lines through the squares represent 95% CIs. The diamonds represent the overall OR from the meta-analyses and the corresponding 95% CIs. An OR greater than 1 implies that the CR event is more likely in the CAG group.

Using historical controls, CAG regimen was compared with non-CAG regimens for AML induction in 7 trials. 165 patients were treated with CAG, 162 were induced with non-CAG regimens (the regimen details are provided in the Table 5). The heterogeneity test of CR event rates from the 7 studies revealed Q 3.631, p 0.726, I^2 ^<0.001, indicating that there was no significant variation among the 7 studies. Therefore, the CR event rates were calculated using the fixed-effects model (Figure 5). Surprisingly, the CR rate of CAG was significantly higher than those of other regimens, with an odds ratio of 2.43 (95% CI, 1.52-3.88) (Figure 5).

**Table 5:** CR rates of CAG and non-CAG regimens in AML patients


**Study**	**Year**	**Total No**. **patients**	**No. Patients**		**No. CR**		**CR %**	
			
			**CAG**	**Non-CAG**	**CAG**	**Non-CAG**	**CAG**	**Non-CAG**

Saito [35]	1995	35	18	17^1^	15	11	83	65

Chen [46]	2008	75	34	41^2^	23	16	68	39

Sang [47]	2008	45	23	22^3^	9	7	39	32

Bian [48]	2008	46	26	26^4^	20	10	77	38

Zhu [50]	2009	50	30	20^5^	14	6	47	30

Li [53]	2010	38	18	20^6^	14	14	78	70

Feng [52]	2010	32	16	16^7^	9	7	56	44

Abbreviation: AML: acute myeloid leukemia; CR: complete remission; CAG: cytarabine, aclarubicin and G-CSF;

1: HD-Ara-C+M/ME (high-dose cytarabine plus mitoxantrone with or without etoposide);

2: TA/HA (pirarubicin plus cytarabine/homoharringtonine plus cytarabine);

3: DA/HA/IA (daunorubicin plus cytarabine/homoharringtonine plus cytarabine/idarubicin plus cytarabine);

4: DAH/MAE (daunorubicin plus cytarabine plus homoharringtonine/mitoxantrone plus cytarabine plus etoposide);

5: DA/IA/MA (daunorubicin plus cytarabine/idarubicin plus cytarabine/mitoxantrone plus cytarabine);

6: HAG (homoharringtonine plus cytarabine plus GCSF);

7: HA (homoharringtonine plus cytarabine).

### Cardiotoxicity and early death rate of CAG

**Figure 6:**
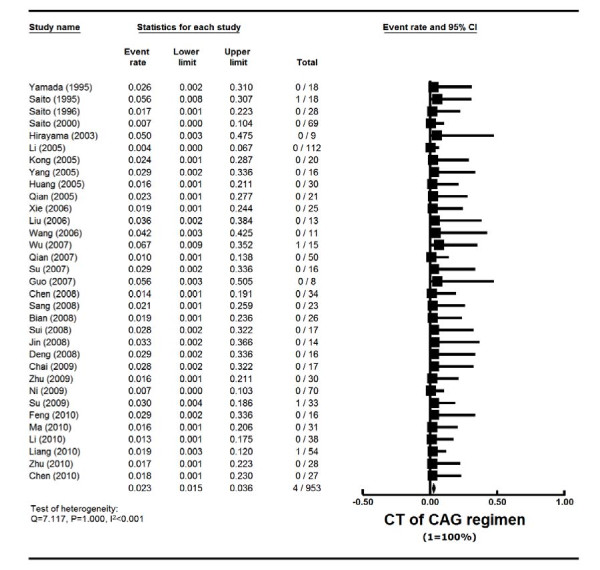
**Cardiotoxicity (CT) rate of CAG regimen in AML and MDS patients--Forest plot of CT event rates**. Summary CT rates of CAG regimen were calculated using the random-effects model. Horizontal lines through the squares represent 95% CIs. The diamonds represent the overall CT event rate from the meta-analyses and the corresponding 95% CIs.

**Figure 7:**
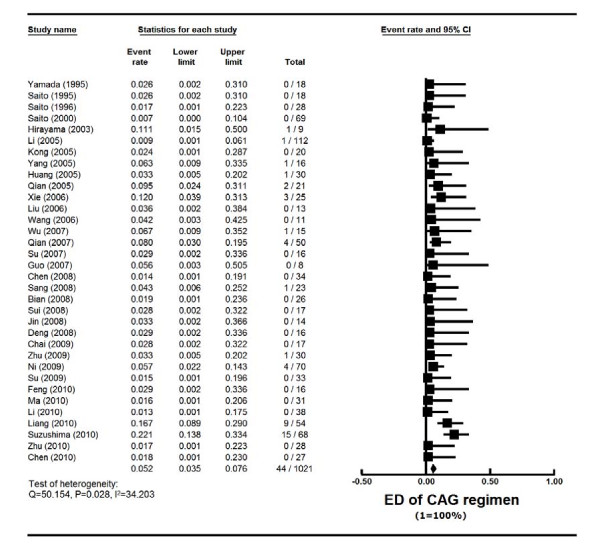
**Early death (ED) rate of CAG regimen in AML and MDS patients**. Summary ED rates of CAG regimen were calculated using the random-effects model. Horizontal lines through the squares represent 95% CIs. The diamonds represent the overall ED event rate from the meta-analyses and the corresponding 95% CIs.

The toxicity of CAG in all reports was generally mild. Cardiotoxicity data were reported in 33 studies with 953 patients, and ED was reported in 34 studies with 1021 patients. Cardiotoxicity rate was 2.3% (95% CI, 1.5%-3.6%) (actuarial rate 0.42%, 4/953) (Tables 1 and 2, Figure 6). ED rate was 5.2% (95% CI, 3.5%-7.6%) (actuarial rate 4.31%, 44/1021) (Tables 1 and 2, Figure 7). Among AML patients, the cardiotoxicity rate was 2.4% (95% CI, 1.5%-4.0%) (actuarial rate 0.41%, 3/738), and ED rate was 5.9% (95% CI, 3.9%-8.8%) (actuarial rate 5.09%, 41/806). Most of the death was caused by severe infection, cardiopulmonary dysfunction or cerebral hemorrhage.

## Discussion

CAG regimen was originally designed in Japan for the treatment of relapsed AML patients in 1995. It quickly became popularized in China for the treatment of high-risk AML and MDS patients due to relatively mild toxicity. Most of the studies were single-center non-randomized trials with small sample size. CAG has not been systematically compared with other induction regimens. In this meta-analysis, 1029 patients with AML and MDS were treated with CAG regimen. Two findings on CR rate for AML are quite intriguing. Number one, there was no significant differences in CR rates between new (56.7%) and relapsed/refractory (60.1%) AML. This is surprising since the CR rate is generally lower for R/R than new AML. One possibility is that this novel CAG regimen can overcome the drug resistance of the R/R AML clone and lead to high CR rate. Alternatively, it may be that the first-line induction therapy was substandard in these patients from diverse institutions with different supportive care standards. Therefore, the relapsed and refractory AML was still sensitive to alternative chemotherapy. The CR rate of CAG (56.7%) for new AML appears to be slightly lower than the CR rate of standard "3+7" regimen (daunorubicin 45 mg/m^2 ^+ Ara-C 100 mg-200 mg/m^2^) (57%-65%) [5,7,30,31] (Table 6). However, there have been no randomized studies, it is therefore not clear whether CAG is as effective as "3+7".

**Table 6:** CR rates, ED and Cardiotoxicity rates of CAG and non-CAG induction regimens in new AML and MDS


**Study (ref)**	**Disease**	**No. Patients**	**Age**	**Median age**	**Regimen**	**CR %**	**ED %**	**CT %**

CALGB 8321 [30]	AML	326	15-83	NA	DNR (45 mg/m^2^) +Ara-C	61	15^1^	NR

ECOG [7]	AML	582	17-60	48	DNR (45 mg/m^2^) +Ara-C DNR (90 mg/m^2^) +Ara-C	57 71	4.5^1^ 5.5^1^	7.2 7.9

SALR [5]	AML	2767	16-97	72	anthracycline + Ara-C	65	10^2^	NR

HOVON and SAKK [31]	AML	813	60-83	67	DNR (45 mg/m^2^) +Ara-C DNR (90 mg/m^2^) +Ara-C	54 64	11^2^ 12^2^	NR

CAG	AML	327	18-88	NA	CAG	57	9.0^3^	2.8

Fenaux [33]	MDS	358	38-88	69	Azacitidine CCR	17 8	11^4^ 9^4^	NR

Kantarjian [34]	MDS	510	17-88	63	TA/FA/CAT/IA/DA	55	17^5^	NR

CAG	MDS	215	26-78	NA	CAG	45.7	4.8^3^	3.1

Abbreviations: AML: acute myeloid leukemia; MDS: myelodysplastic syndrome; Ref: references; NR: not reported; CR: complete remission; NA: not available; ED: early death; CT: cardiotoxicity; DNR: daunorubicin; Ara-C: cytarabine; 3+7: anthracycline plus cytarabine; CAG: cytarabine, aclarubicin and G-CSF; CCR: conventional care regimen; TA: Topotecan plus Ara-C; FA: Fludarabine plus Ara-C; CAT: Topotecan plus Ara-C plus cyclophosphamide; DA/IA: daunorubicin or idarubicin plus Ara-C; CALGB: Cancer and Leukemia Group B; ECOG: Eastern Cooperative Oncology Group; SALR: Swedish Acute Leukemia Registry; HOVON: Leukemia Working Group of the Dutch-Belgian Cooperative Trial Group for Hematology-Oncology; SAKK: Swiss Group for Clinical Cancer Research;

Superscript numerical indicates the definitions of early death from different studies. 1: induction mortality (time frame not specified); 2: mortality within 30 days from diagnosis; 3: mortality within the first 8 weeks of induction treatment; 4: mortality during first 3 months; 5: mortality within 7 weeks of induction therapy.

The second finding is that, as frontline AML regimen, CAG induced higher CR rate in AML than non-CAG regimens (p < 0.001, odds ratio 2.43 favoring CAG). The non-CAG regimens included anthracyclines plus Ara-C in 5 studies (AA regimens), and homoharringtonine plus Ara-C (HA regimen) in two other studies. HA is only used in China [32]. When CAG was compared with the 5 AA regimens, CAG remains superior to the AA regimens in CR rate (p < 0.001, odds ratio 2.73, 95% CI 1.61-4.64). However, the studies were not randomized, and the comparisons in those studies were made with historical controls. It is therefore worthwhile to compare CAG regimen with standard anthracycline plus Ara-C in a prospective randomized study.

In this meta-analysis, the CR rate of CAG regimen for high-risk MDS/t-AML patients was 45.7% (95% CI, 39.0%-52.4%). Aza-001 trial, a prospective randomized study, compared azacitidine with conventional care regimen (best supportive care, low dose Ara-C, or intensive chemotherapy "3+7") in higher-risk MDS patients [33]. The CR rate was 17% and 36% in the azacitidine group and "3+7" group, respectively. Yet azacitidine led to prolonged overall survival in the high-risk MDS patients. CAG gained popularity in China and Japan in the past decade because, based solely on small phase II studies, it is widely believed to be a milder regimen than "3+7" for high-risk and elderly AML and MDS patients. It is therefore valuable to perform a prospective randomized trial in high-risk elderly MDS patients to compare CAG with "3+7" for their effects on overall survival.

There have not been many reports focusing on toxicities of AML inductions. In the report of "3+7" regimen on 326 new AML patients from the Cancer and Leukemia Group B 8321 study, the overall induction mortality was 15% [30]. Data from the Swedish Acute Leukemia Registry from all unselected 2767 AML patients found an overall ED (30 days from diagnosis) rate of 19% (range 4-40% according to age) [5]. The ED rate was 10% for intensive chemotherapy group, and 34% for palliative group. The data clearly supported chemotherapy for all age groups. In a phase III randomized study comparing high with standard dose daunorubicin in 657 young (age ≤ 60) AML patients [7], induction mortality was 4.5% in the standard group, 5.5% in the high dose group (p = 0.60). This study reported cardiac toxicity of 7.2% in the standard dose group with no reduction in ejection fraction. In a separate phase III randomized study comparing high with standard dose daunorubicin in 813 elderly (age ≥ 60) AML patients [31], 30-day mortality was found to be 12% in the standard group, 11% in the high dose group (p = 0.59). Cardiac toxicity was not specified in this study. Kantarjian's group reported their 15 years' experience of induction chemotherapy on 510 high-risk MDS patients (median age 63) [34]. The overall ED (death within 7 weeks of induction) rate was 17% (topotecan-Ara-C 6%, anthracycline-Ara-C 17%, fludarabine-Ara-C 23%). In our analysis, it was difficult to quantify toxicities from so many small phase II studies, which were from diverse institutions in China and Japan with different supportive care standards. We therefore chose two relatively objective parameters, cardiotoxicity and ED rate. In this analysis, cardiotoxicity was 2.3% and ED was 5.2% among all AML and MDS patients. For new AML patients, the ED rate was 9.0%, whereas the ED for MDS was only 4.8% (Table 6). Taken together all these experiences of induction efficacy and toxicities (Table 6), it appears that CAG regimen was effective and well tolerated.

This study has severe limitations. First of all, this study is based on 35 trials which were conducted in China and Japan, where significant variations in supportive care may have existed. In addition, the studies were between 1995 and 2010. Newer molecular prognostic markers, such as FLT3 and NPM1, were not routinely tested in most of the studies. The standard of supportive care for AML has changed dramatically over the decade. The toxicity data from this analysis may therefore overestimate the adverse events of the regimens under current health care system. Majority of the studies were small (only 9 of the 35 studies had 50 or more subjects). Although all the studies used similar CAG regimens, the dosage of aclarubicin was variable. Due to the small sample sizes and ambiguity in reporting the AML status in the 7 studies, it was difficult to ascertain whether the variation of the aclarubicin dosage could have played any role in the outcomes among the different groups of AML (new vs R/R) and MDS/t-AML. There was no randomized study among the 35 reports. CAG was compared with non-CAG regimens from historical controls in the 7 reports. The non-CAG regimens also varied. Two of the seven reports did not include anthracyclines. It is therefore possible that the superiority of CAG over non-CAG regimen may have been overestimated, even when the more stringent fixed-effects model was used. Estimation of CR event rates using random-effects model may minimize the inherent variances. Finally, CAG regimen is used exclusively in China and Japan. It is not clear whether similar outcome is reproducible in the Western countries.

In conclusion, CAG regimen was effective and safe for the treatment of AML and MDS patients. Its activity may vary among these patients with significantly higher CR rates observed in AML than MDS. It was as active in new AML as in relapsed and refractory AML. CAG regimen may be more effective than non-CAG regimen with the CR rate of CAG regimen significantly higher than those of non-CAG induction regimens in AML patients. This regimen was well tolerated with low cardiotoxicity and ED rate. We strongly recommend that CAG regimen be compared with standard anthracycline plus Ara-C in a prospective randomized study, particularly in high-risk and elderly AML and MDS patients.

## Competing interests

The authors declare that they have no competing interests.

## Authors' contributions

GW and DL participated in concept design, data collection and analysis, drafting and critically revising the manuscript. All authors are involved in reviewing and revising the manuscript. All authors have read and approved the final manuscript.
